# The Impact of the Metabolic Syndrome and Its Components on Resting Energy Expenditure

**DOI:** 10.3390/metabo12080722

**Published:** 2022-08-05

**Authors:** Mario Soares, Yun Zhao, Emily Calton, Kaveri Pathak, Wendy Chan She Ping-Delfos, Nicola Cummings, Patience Nsatimba

**Affiliations:** 1 School of Population Health, Curtin University, Perth, WA 6102, Australia; 2 Children’s Diabetes Centre, Telethon Kids Institute, Perth, WA 6009, Australia; 3 WA Eating Disorder Specialist Service, Mental Health, Public Health and Dental Services, Perth, WA 6003, Australia; 4 Nursing Directorate, Pantang Hospital, Greater Accra, Accra GA184, Ghana

**Keywords:** resting energy expenditure, metabolic syndrome, insulin sensitivity, metabolic rate

## Abstract

We determined whether metabolic syndrome (MetS) and the increasing number of its components influenced the resting energy expenditure (REE). Data on adult men (*n* = 72, 40%) and women (*n* = 108, 60%) from European (*n* = 154, 86%) and Sub-Saharan African (*n* = 26, 14%) ancestry were used. Ninety-five (53%) participants had MetS (MetS+), while 85 (47%) were without MetS (MetS−). REE was determined through indirect calorimetry, body composition by DEXA, and clinical biochemistry by standard laboratory techniques. MetS+ had a significantly higher REE (mean ± se: MetS+: 5995 ± 87.3 vs. MetS−: 5760 ± 86.3 kJ/d, *p* = 0.025) when adjusted for age, gender, fat mass (FM), fat-free mass (FFM), ethnicity, season, 25OHD, insulin sensitivity, and time of data collection. Within each MetS status group, an increase in the number of components (C) resulted in a stepwise increase in REE. Relative to zero components, those with 1C had adjusted REE higher by +526 ± 248.1 kJ/d (*p* = 0.037), while 2C were higher than 1C by +298 ± 140.8 kJ/d (*p* = 0.037). Similarly, relative to 3C, those with 4C had REE higher by +242 ± 120.7 kJ/d (*p* = 0.049). The higher REE of 5C over 4C by 132 ± 174.5 kJ/d did not achieve statistical significance. MetS was associated with a significantly higher REE. This greater energetic cost varied directly with the numbers of its components but was most evident in those not diagnosed with the syndrome.

## 1. Introduction

Measures of total energy expenditure (TEE) rather than energy intake are used to determine human energy requirements. The latter varies with age, gender, body size, physical activity, and physiological status [1,2,3,4]. Resting energy expenditure (REE) makes up ~70% of TEE in sedentary individuals and hence forms the basis of estimating energy requirements [4]. On accounting for age, gender, fat mass (FM), and fat-free mass (FFM), there is ~15% residual variability in REE that has not been defined [4]. Various authors have indicated that part of this residual inter-individual variability may be explained through either detailed body composition that includes organ tissue masses and skeletal muscle mass [4,5,6,7,8], a variety of hormones (thyroid, leptin, cortisol, adiponectin, etc.) [9,10] and possibly adipokines [11]. We have reported that prevailing vitamin D status, as measured by 25 hydroxyvitamin D (25OHD), as well as insulin sensitivity (IS), also made significant contributions to residual variability in REE [12]. While 25OHD was positively related to REE, IS had a negative association with REE [12]. We reconciled those observations through a model that proposed a direct positive pathway of 25OHD onto REE, and a negative mediatory pathway of a variety of insulin sensitivity/insulin resistance (IS/IR) indices, on the link between 25OHD and REE [13].

The prevalence of metabolic syndrome (MetS) is globally very high, and this metabolic derangement increases the risk of cardiovascular disease (CVD) and type 2 diabetes (T2DM). Insulin resistance and low-grade chronic inflammation may underscore the evolution of this syndrome, which is exacerbated by increasing adiposity [11]. A higher energy cost of MetS may be expected due to its links to cellular inflammation and enhanced immune cell activation, which are energetically expensive [14]. In this paper, we determined whether MetS per se or increases in the number of its components would be associated with an elevated REE. Since vitamin D status may improve IS [15,16,17,18] and lessen MetS [19,20], any putative influence of the presence of MetS on REE would need to control for the effects of both vitamin D and IS.

## 2. Materials and Methods

The data on 180 adult Australians of two ethnic groups (Caucasian, *n* = 154; sub-Saharan Africans (SSA), *n* = 26) is used for this analysis. The details of subjects and recruitment have been detailed before [13,21] but are also briefly indicated here. REE was measured by canopy mode indirect calorimetry (*n* = 144; Deltatrac II, Datex Instrumentarium, Finland or *n* = 36 TrueOne, Parvo Medics, Salt Lake city, UT, USA), using a standardized protocol that emphasized a 10–12 h overnight fast, 24 h abstinence from heavy physical activity, and a mandatory 30 min rest in the supine position prior to measurement [12,13,21]. The TrueOne provides excellent CVs for accuracy and reliability and has been validated against the Deltatrac II [22,23]. All measurements were conducted between 22–25 °C in a temperature-controlled room. Minute-to-minute recordings of O_2_ consumption and CO_2_ production were made over 30 min. Weir’s equation calculated REE from the average of the last 25 minutes of data collection [24]. The respiratory quotient (RQ) was measured by dividing CO_2_ production by O_2_ consumption over the same period. None of the participants in this collation had a measured RQ < 0.7 or >1.0 [25]. Body composition was assessed using dual-energy X-ray absorptiometry (DEXA, DPX-L (*n* = 28) or Prodigy Models (*n* = 155), Lunar Corporation, USA), and a validation study has demonstrated equivalence in their estimates [26]. Fasting blood clinical chemistry measurements were conducted by the accredited laboratory of the Department of Pathology, Royal Perth Hospital, Perth, WA. Vitamin D status (25OHD) was determined using the chemiluminescence immunoassay method (*n* = 69 Liaison, DiaSorin or *n* = 114 Architect, Abbott).

The components of metabolic syndrome in each individual include waist circumference, fasting glucose, triglycerides, high-density lipoprotein, and resting systolic or diastolic blood pressure [27]. Individuals presenting with values higher than the recommended cut-off for three or more components were diagnosed with metabolic syndrome (MetS+) [27], while those with <3 were designated as MetS−. Several surrogate markers exist for IS and IR in both the fasting and postprandial states [28], and represent mathematical expressions that include insulin, glucose, or triglycerides (TG). As glucose and TG are components of MetS, we decided to use a more straightforward index based on the inverse natural log of insulin (Inv_IN). The relationship of this marker to other commonly used markers like McAuley’s insulin sensitivity index (McA) [28] and Quantitative insulin check index (QUICKI) [28] was tested prior to the application of Inv_IN.

### 2.1. Participant Selection & Ethical Standards

Participants in this analysis identified as of European ancestry or sub-Saharan African descent and had been resident in Perth, WA for >2 years; were aged between 19 to 80 years, with a body mass index (BMI) of mean ± sd 29.9 ± 5.7 kg/m^2^. The detailed inclusion and exclusion criteria have been reported previously [13] but briefly included weight stability, not on medications that affect metabolism or body composition, and no clinical diagnosis of chronic disease. Data were collected across two time points, 2004–2008 and 2013–2017, and this coincided with changes in REE instrument used and in 25OHD assays. To account for these changes in methodology, we created and adjusted for a categorical variable’ time of measure’ in this analysis.

### 2.2. Statistical Analysis

The primary outcome of this study was REE, and the key variable of interest was MetS status and its components. Nine covariates were chosen based on our experience, a literature review, and the availability of variables in all participants in this study. With 80% power at a 5% significance level, we estimated a sample size of 170 was required to detect a medium effect size (0.28) for the variable of interest, MetS and its components, using analysis of covariance (ANCOVA) with nine covariates (GPower version 3.1) [29]. The dataset we used in this paper had a sample size of 180 with no missing values, and this estimate also satisfied the recommendations of Green [30].

**Figure 1:**
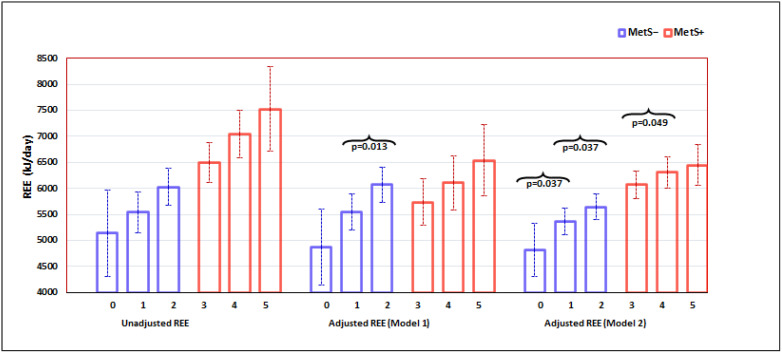
The impact of an increasing number of MetS components on REE in adults with and without MetS. Vertical bars are mean REE with a 95% confidence interval. Unadjusted REE: Tested by one-way ANOVA. Adjusted REE: Tested by General Linear Univariate model. Model 1: adjusted for age, gender, season, time of measure, and ethnicity. Model 2: adjusted for those in Model 1 plus FM, FFM, 25OHD & insulin sensitivity.

Continuous (assessed for normality) and categorical variables were reported as mean (standard deviation) or median (IQR) for skewed variables and frequency (%), respectively. Independent samples t-test (or Mann-Whitney U test for non-normally distributed data) and chi-square test was used for examining the differences between MetS status (MetS− = without MetS; MetS+ = with MetS) for continuous and categorical variables, respectively. Analysis of covariance (ANCOVA) (via General Linear Univariate model function) was conducted to assess the association between MetS status (and its components) and REE, with an adjustment of covariates or confounders through two models. In Model 1, we controlled for age (year), gender (0 = women, 1 = men), season (0 = winter/spring, 1 = summer/autumn), time of data collection (0 = 2004–2008; 1 = 2013–2017), and ethnic group (0 = South African, 1 = European). In Model 2 we further adjusted for fat mass (kg), fat-free mass (kg), vitamin D status (25OHD) at the start of the study, and insulin sensitivity in addition to those already being controlled in Model 1. Statistical significance was set at a *p*-value less than 0.05. Post-hoc tests employed the Fisher’s Least Significant Difference (LSD) procedure as we only compared two categories in each MetS group (Figure 1). All analyses were performed by using SPSS (IBM Corp. Released 2019. IBM SPSS Statistics for Windows, Version 26.0. IBM Corp.: Armonk, NY, USA.

## 3. Results

The general characteristics of the MetS groups are compared in Table 1. Those with MetS+ had significantly higher values for WC, FPG, TG, SBP, and DBP, while HDL-C was lower (Table 1). MetS+ also had higher BMI, FM, FFM, and REE. Pearson correlations between Inv_IN used in this analysis and other commonly used surrogate markers for IS were: McAuley’s index, r = 0.72; *p* < 0.001 and QUICKI: r = 0.866; *p* < 0.001. Inv_IN was also significantly related to FM (r = −0.34, *p* < 0.001), REE (−0.37, *p* < 0.001) and 25OHD (r = 0.23, *p* = 0.002); all in the same direction as McAuley’s index and QUICKI (data not shown).

Adjusted for covariates, on average, the MetS+ group still had a higher REE (Table 2). For both MetS groups, we observed a positive dose–response relationship between REE and the number of components, demonstrating that increased REE was associated with an increased number of components (Figure 1). In the fully adjusted Model 2 (Figure 1), for the MetS− group, we noted that individuals with one component had a significantly higher REE (5369.1 ± 129.4 kJ/d) on average compared to those who were normal with nil components (4842.8 ± 260.1 kJ/d; an increase by 526 ± 248 kJ/d, *p* = 0.037). Individuals with two components had a higher mean REE (5667.0 ± 125.1 kJ/d) compared to those with one component (increase by 298 ± 141 kJ/d, *p* = 0.037). Figure 1 further shows that the REE of MetS− individuals with two components was higher by 824 kJ/d over those with nil (0) components (*p* = 0.003).

Similarly, in the MetS+ group, participants with four components had a significantly higher mean REE (6309.4 ± 150.5 kJ/d) than those with three components (6067.9 ± 135.4 kJ/d); an increase of 241 ± 121 kJ/d, *p* = 0.049). The mean REE of participants with five components (6441.5 ± 196.6 kJ/d) was also higher than that for those with four components with a sizeable difference (132 ± 175 kJ/d) which, however, did not achieve statistical significance. In addition, Figure 1 shows that overall, the mean difference in REE between those MetS+ individuals with five vs. three components was 374 kJ/d (*p* = 0.041).

## 4. Discussion

Metabolic syndrome is defined by a combination of anthropometric, metabolic, and hemodynamic abnormalities that can congregate in any individual [27]. The global rise in obesity rates has increased the incidence of MetS, with a flow-through effect on type 2 diabetes mellitus (T2DM) and cardiovascular disease (CVD). Weight loss and maintenance are key to reducing MetS in the community and the clinic/hospital setting. The prescription of a caloric deficit aimed at weight loss begins with the determination of REE. While direct measurements of REE are best, most clinical dietitians/nutritionists would predict REE. To date, no global generalized equations in the literature allow MetS to predict REE. This analysis aimed to determine whether metabolic syndrome (MetS), or increases in the number of its components, elevated REE. Body composition is a key determinant of REE and, depending on the model of investigation used, may account for up to 80% of the variation in REE [4]. The MetS+ group had higher BMI and was significantly heavier due to greater FM and FFM (Table 1). They were also more insulin resistant (Table 1). On adjusting for these differences and other covariates, we observed that REE was significantly greater in those with MetS (Table 2, model 2). REE showed a pattern suggestive of a stepwise rise with each increase in the number of MetS components (Figure 1, model 2). This may indicate an energy cost associated with each component of MetS. Interestingly, in MetS− participants, we observed an elevated REE associated with the presence of even one or two components (Figure 1, model 2), and the overall increase in REE from zero to two components was +824 kJ/d; more than double that seen from three to five components (+374 kJ/d; Figure 1, model 2).

The precise mechanisms for the higher REE in MetS are still unknown and could vary with each component under investigation. Chronic low-grade inflammation with activated immune cell function is energetically expensive [31]. Previous studies have shown that individuals with glucose intolerance have a higher REE compared to normal subjects [32], with fasting hyperglycemia predictive of lower rates of weight gain by increased energy expenditure and fat oxidation rate [32]. Wahlqvist et al. [33] proposed that energy dysregulation may underscore MetS in their study of a large cohort of ethnically diverse US and Taiwan population groups. They reported that waist, glucose, and triglycerides formed a homogenous cluster across all ethnic groups and that these factors determined the greatest variance in MetS. Waist circumference reflects visceral adiposity—a metabolically active compartment—which through increased free fatty acid (FFA) flux, affects hepatic metabolism of glucose and triglycerides [34]. Several studies on ethnically diverse adults report a positive link between blood pressure and REE [35,36,37]. A small but significant proportion of REE is determined by the basal activity of the sympathetic nervous system (SNS) [38,39]. The SNS is a significant player in the neuro-humoral control of blood pressure [40] and has been shown to be elevated in hypertension [41]. There is some evidence that SNS activity may explain the link between REE and SBP observed in this study [42,43]. Following caloric restriction, the REE adjusted for change in body composition was significantly lower in those who recovered from MetS relative to those who did not [44]. Interestingly, a sizeable proportion of that change in REE was accounted for by changes in TG and an interaction of change in glucose x gender [44]. Such outcomes would support the contention of Wahlqvist et al. [33]. REE is a fundamental requirement for the energy of all respiring tissues at rest, and it closely parallels measures of mitochondrial function [45]. It has been suggested that the ethnic propensity for a chronic disease may be underscored by mitochondrial dysfunction [46]. Accordingly, mitochondrial DNA polymorphisms and/or alterations in function could theoretically induce variations in ATP formation and heat generation (i.e., uncoupling) that confer an increased risk of disease when the environment changes. Collectively, there is good evidence in support of an energetic cost to MetS, as proposed in this paper.

## 5. Strengths & Weaknesses

The overall sample size was adequate, but as we did not select for a number of components, both the zero and five component groups had fewer participants (Table 1). This was particularly true of the SSA group, younger than the European group, which mainly had one or two MetS components and contributed none to other groups (Figure 1). Consequently, some interaction terms could not be tested in our statistical models. We also cannot comment on the possible moderation by ethnicity in the relationship between MetS and REE, and the latter would serve as an interesting avenue for future work. However, the dataset provides gold standard methods of measurement for REE and body composition, well-conducted protocols, and a list of key covariates -both biological and methodological- that allowed a truer determination of the nexus between MetS and REE.

## 6. Conclusions

There is a significant energy cost associated with MetS, which increases in a stepwise manner with the number of components. These added costs were, overall, of a greater magnitude (~800 kJ/d) in those without MetS. Validation of these findings would confirm the influence of MetS on energy metabolism.

## Figures and Tables

**Table 1:** Demographic and metabolic characteristics of the study population.

Variable	MetS− *n* = 95	MetS+ *n* = 85	*p* Value *
Age, years	41.4 ± 14.7	55.3 ± 10.5	0.001
Gender (*n*, %) Female Male	64 (67.4) 31 (32.6)	44 (51.8) 41 (48.2)	0.033
Ethnicity (*n*, %) Sub- Saharan African European	21 (22.1) 74 (77.9)	5 (5.9) 80 (94.1)	0.002
Season (*n*, %) Winter/spring Summer/autumn	63 (66.3) 32 (32.7)	39 (45.9) 46 (54.1)	0.006
Time of data collection (*n*, %) 2004–2008 2013–2017	20 (21.1) 75 (78.9)	49 (57.6) 36 (42.4)	0.001
BMI, kg/m^2^ Fat mass, kg	27.2 ± 5.15 27.7 ± 11.2	32.9 ± 4.89 37.2 ± 10.3	0.001 0.001
Fat-free mass, kg	50.5 ± 10.9	57.4 ± 12.4	0.001
Total MetS components (*n*, %) 0 1 2 3 4 5	9 (9.5) 38 (40.0) 48 (50.5) n.a n.a n.a	n.a n.a n.a 44 (51.8) 31 (36.5) 10 (11.8)	0.001
WC, cm	91.1 ± 13.6	106.0 ± 11.9	0.001
FPG, mmol/L	5.2 ± 0.49	6.2 ± 0.88	0.001
TG, mmol/L	1.07 (0.51)	2.04 (1.1127)	0.001
HDL-C, mmol/L	1.85 (0.786)	1.31 (0.499)	0.001
SBP, mmHg	120 ± 13.4	133 ± 14.4	0.001
DBP, mmHg	71 ± 8.7	79.0 ± 8.8	0.001
Inv_IN	0.606 (0.187)	0.496 (0.162)	0.001
25OHD nmol/L	60.6 ± 24.08	57.2 ± 18.57	0.293

*n* = 180. Data are mean ± s.d for continuous variables and *n* (%) for categorical variables. MetS− without MetS; MetS+ = with MetS; BMI, body mass index; WC, waist circumference; FPG, fasting plasma glucose; TG, triglycerides; HDL-C, high-density lipoprotein; SBP, systolic blood pressure; DBP, diastolic blood pressure; Inv_IN = inverse LN insulin; * Independent samples t-test for continuous variables or chi-square test for categorical variables; *p* < 0.05 denotes statistical significance. n.a, not applicable as MetS is defined as the presence of 3 or more metabolic derangements.

**Table 2:** The contribution of metabolic syndrome to REE of adult men and women.

	MetS−	MetS+	* *p* Value
Unadjusted REE, kJ/d	5781.4 ± 132.9	6814.7 ± 140.5	<0.001
Adjusted REE, kJ/d (Model 1)	5408.9 ± 135.6	6283.8 ± 138.9	<0.001
Adjusted REE, kJ/d (Model 2)	5760.2 ± 86.3	5994.1 ± 87.3	0.025

*n* = 180. Data are mean ± standard error. MetS− = without metabolic syndrome; MetS+ = with metabolic syndrome. Using pooled data (over MetS− and MetS+) and *p* Value: * Independent samples t-test or Multivariable linear regression analysis (via General Linear Univariate model). LSD test was used for post-hoc comparison. Model 1: adjusted for age, gender, season, time of measure, and ethnicity. Model 2: adjusted for model 1 plus FM, FFM, 25OHD & insulin sensitivity.

## Data Availability

The data used in this paper is freely available to any researcher wishing to use them for non-commercial purposes, provided the institution’s human ethics committee approves the request.

## References

[1] Donahoo W.T., Levine J.A., Melanson E.L. (2004). Variability in energy expenditure and its components. Curr. Opin. Clin. Nutr. Metab. Care.

[2] Goran M.I., Nagy T.R., Gower B.A., Mazariegos M., Solomons N., Hood V., Johnson R. (1998). Influence of sex, seasonality, ethnicity and geographic location on the components of total energy expenditure in young children: Implications for energy requirements. Am. J. Clin. Nutr..

[3] Westerterp K.R. (2017). Control of energy expenditure in humans. Eur. J. Clin. Nutr..

[4] Soares M.J., Müller M.J. (2018). Resting energy expenditure and body composition: Critical aspects for clinical nutrition. Eur. J. Clin. Nutr..

[5] Garby L., Lammert O. (1994). Between-subject variation in energy expenditure: Estimation of the effect of variation in organ size. Eur. J. Clin. Nutr..

[6] Piers L.S., Soares M.J., McCormack L.M., O’Deaa K. (1998). Is there evidence for an age-related reduction in metabolic rate?. J. Appl. Physiol..

[7] Illner K., Brinkmann G., Heller M., Bosy-Westphal A., Müller M.J. (2000). Metabolically active components of fat-free mass and resting energy expenditure in non-obese adults. Am. J. Physiol. Endocrinol. Metab..

[8] Löffler M.C., Betz M.J., Blondin D.P., Augustin R., Sharma A.K., Tseng Y.H., Scheele C., Zimdahl H., Mark M., Hennige A.M. (2021). Challenges in tackling energy expenditure as obesity therapy: From preclinical models to clinical application. Mol. Metab..

[9] Al Adsani H., Hoffer L.J., Silva J.E. (1997). Resting energy expenditure is sensitive to small dose changes in patients on chronic thyroid hormone replacement. J. Clin. Endocrinol. Metab..

[10] Soares M.J., Piers L.S., O’Deaa K., Collier G.R. (2000). Plasma leptin concentrations, basal metabolic rates and respiratory quotients in young and older adults. Int. J. Obes. Relat. Metab. Disord..

[11] Sepandar F., Rashidbeygi E., Maghbooli Z., Khorrami-Nezhad L., Hajizadehoghaz M., Mirzaei K. (2019). The association between resting metabolic rate and metabolic syndrome May Be mediated by adipokines in overweight and obese women. Diabetes Metab. Syndr. Clin. Res. Rev..

[12] Calton E.K., Pathak K., Soares M.J., Alfonso H., Keane K.N., Newsholme P., Cummings N.K., Chan She Ping-Delfos W., Hamidi A. (2016). Vitamin D status and insulin sensitivity are novel predictors of resting metabolic rate: A cross-sectional analysis in Australian adults. Eur. J. Nutr..

[13] Soares M.J., Calton E.K., Pathak K., Zhao Y. Hypothesized Pathways for the Association of Vitamin D Status and Insulin Sensitivity with Resting Energy Expenditure: A Cross Sectional Mediation Analysis in Australian Adults of European Ancestry. Eur. J. Clin. Nutr..

[14] Keane K.N., Calton E.K., Cruzat V.F., Soares M.J., Newsholme P. (2015). The impact of cryopreservation on human peripheral blood leucocyte bioenergetics. Clin. Sci. Lond..

[15] von Hurst P.R., Stonehouse W., Coad J. (2010). Vitamin D supplementation reduces insulin resistance in South Asian women living in New Zealand who are insulin resistant and vitamin D deficient-a randomised, placebo-controlled trial. Br. J. Nutr..

[16] Belenchia A.M., Aneesh K.T., Laura S.H., Peterson C.A. (2013). Correcting vitamin D insufficiency improves insulin sensitivity in obese adolescents: A randomized controlled trial. Am. J. Clin. Nutr..

[17] Theodoratou E., Tzoulaki L., Zgaga L., Ioannidis J.P.A. (2014). Vitamin D and multiple health outcomes: Umbrella review of systematic reviews and meta-analyses of observational studies and randomised trials. BMJ.

[18] Pathak K., Soares M.J., Zhao Y., James A.P., Sherriff J.L., Newsholme P. (2017). Postprandial changes in glucose oxidation and insulin sensitivity in metabolic syndrome: Influence of fibroblast growth factor 21 and vitamin D status. Nutrition.

[19] Pannu P.K., Soares M.J., Piers L.S., Zhao Y., Ansari Z. (2017). The association of vitamin D status and dietary calcium intake with individual components of the metabolic syndrome: A population based study in Victoria, Australia. Cardiovasc. Endocrinol..

[20] Soares M.J., Pannu P.K., Calton E.K., Hills A.P., Read C.J. (2017). Vitamin D status and Calcium intake in systemic inflammation, insulin resistance and the metabolic syndrome: An update on current evidence. Trends Food Sci. Technol..

[21] Nsatimba A., Pathak K., Soares M.J. (2016). Ethnic differences in resting metabolic rate, respiratory quotient and body temperature: A comparison of Africans and European Australians. Eur. J. Nutr..

[22] Cooper J.A., Watras A.C., O’Brienn M.J., Luke A., Dobratz J.R., Earthman C.P., Schoeller D.A. (2009). Assessing validity and reliability of resting metabolic rate in six gas analysis systems. J. Am. Diet. Assoc..

[23] Kaviani S., Schoeller D.A., Ravussin E., Melanson E.L., Henes S.T., Dugas L.R., Dechert R.E., Mitri G., Schoffelen P.F., Gubbels P. (2018). Determining the accuracy and reliability of indirect calorimeters utilizing the methanol combustion technique. Nutr. Clin. Pract..

[24] Weir J.B. (1990). New methods for calculating metabolic rate with special reference to protein metabolism. Nutrition.

[25] Compher C., Frankenfield D., Keim N., Roth-Yousey L. (2006). Best practice methods to apply to measurement of resting metabolic rate in adults: A systematic review. J. Am. Diet. Assoc..

[26] Hull H., He Q., Thornton J., Javed F., Allen L., Wang J., Pierson R.N., Gallagher D. (2009). iDXA, Prodigy, and DPXL dual-energy X-ray absorptiometry whole-body scans: A cross-calibration study. J. Clin. Densitom..

[27] Alberti K.G. (2009). Harmonizing the metabolic syndrome: A joint interim statement of the International Diabetes Federation Task Force on Epidemiology and Prevention; National Heart, Lung, and Blood Institute; American Heart Association; World Heart Federation; International Atherosclerosis Society; and International Association for the Study of Obesity. Circulation.

[28] Lorenzo C., Haffner S.M., Stancakova A., Laakso M. (2010). Relation of direct and surrogate measures of insulin resistance to cardiovascular risk factors in nondiabetic finnish offspring of type 2 diabetic individuals. J. Clin. Endocrinol. Metab..

[29] Faul F., Erdfelder E., Buchner A., Lang A.G. (2009). Statistical power analyses using G*Power 3.1: Tests for correlation and regression analyses. Behav. Res. Methods.

[30] Green S. (1991). How many subjects does it take to do a regression analysis. Multivar. Behav. Res..

[31] Straub R.H. (2017). The brain and immune system prompt energy shortage in chronic inflammation and ageing. Nat. Rev. Rheumatol..

[32] Piaggi P., Thearle M.S., Bogardus C., Krakoff J. (2015). Fasting hyperglycemia predicts lower rates of weight gain by increased energy expenditure and fat oxidation rate. J. Clin. Endocrinol. Metabol..

[33] Wahlqvist M.L., Chang H.Y., Chen C.C., Hsu C.C., Chang W.C., Wang W.S., Hsiung C.A. (2010). Is impaired energy regulation the core of the metabolic syndrome in various ethnic groups of the USA and Taiwan?. BMC Endocr. Disord..

[34] Rui L. (2014). Energy metabolism in the liver. Compr. Physiol..

[35] Luke A., Adeyemo A., Kramer H., Forrester T., Cooper R.S. (2004). Association between blood pressure and resting energy expenditure independent of body size. Hypertension.

[36] Creber C., Cooper R.S., Plange-Rhule J., Bovet P., Lambert E.V., Forrester T.E., Schoeller D., Riesen W., Korte W., Cao G. (2018). Independent association of resting energy expenditure with blood pressure: Confirmation in populations of the African diaspora. BMC Cardiovasc. Disord..

[37] Ali N., Mahmood S., Manirujjaman M., Perveen R., Al Nahid A., Ahmed S., Khanum F.A., Rahman M. (2017). Hypertension prevalence and influence of basal metabolic rate on blood pressure among adult students in Bangladesh. BMC Public Health.

[38] Monroe M.B., Seals D.R., Shapiro L.F., Bell C., Johnson D., Jones P.P. (2001). Direct evidence for tonic sympathetic support of resting metabolic rate in health adult humans. Am. J. Physiol. Endocrinol. Metab..

[39] Welle S., Schwartz R.G., Statt M. (1991). Reduced metabolic rate during beta-adrenergic blockade in humans. Metabolism.

[40] Valensi P. (2021). Autonomic nervous system activity changes in patients with hypertension and overweight: Role and therapeutic implications. Cardiovasc. Diabetol..

[41] Seravalle G., Grassi G. (2016). Sympathetic nervous system, hypertension, obesity and metabolic syndrome. High Blood Press. Cardiovasc. Prev..

[42] Sharma A.M., Pischon T., Hardt S., Kunz I., Luft F.C. (2001). Hypothesis: β-adrenergic receptor blockers and weight gain: A systematic analysis. Hypertension.

[43] Bélanger M., Boulay P. (2005). Effect of an aerobic exercise training program on resting metabolic rate in chronically beta-adrenergic blocked hypertensive patients. J. Cardiopulm. Rehabil. Prev..

[44] Soares M.J., Cummings N.K., Ping-Delfos W.L. (2011). Energy metabolism and the metabolic syndrome: Does a lower basal metabolic rate signal recovery following weight loss?. Diabetes Metab. Syndr. Clin. Res. Rev..

[45] Larsen F.J., Schiffer T.A., Sahlin K., Ekblom B., Weitzberg W., Lundberg J.O. (2011). Mitochondrial oxygen affinity predicts basal metabolic rate in humans. FASEB.

[46] Bhopal R.S., Rafnsson S.B. (2009). Could mitochondrial efficiency explain the susceptibility to adiposity, metabolic syndrome, diabetes and cardiovascular diseases in South Asian populations?. Int. J. Epidemiol..

